# Complementary medicine usage in surgery: a cross-sectional survey in Germany

**DOI:** 10.1186/s12906-022-03746-3

**Published:** 2022-10-11

**Authors:** Ann-Kathrin Lederer, Yvonne Samstag, Thomas Simmet, Tatiana Syrovets, Roman Huber

**Affiliations:** 1 Center for Complementary Medicine, Department of Medicine II, Faculty of Medicine, Medical Center-University of Freiburg, University of Freiburg, Hugstetter Straße 55 – Haus Frerichs, 79106 Freiburg, Germany; 2 Department of General, Visceral and Transplantation Surgery, University Medical Center, Mainz, Germany; 3 Institute of Immunology, Section Molecular Immunology, University Hospital of Heidelberg, Heidelberg, Germany; 4 Institute of Pharmacology of Natural Products & Clinical Pharmacology, Ulm University, Ulm, Germany

**Keywords:** Complementary therapies, Survey, Questionnaire, Health knowledge, Attitudes

## Abstract

**Background:**

Complementary medicine (CM) is frequently used by patients, but little is known about the usage of CM in surgical patients. The study aimed to elucidate the relevance of CM in surgery.

**Methods:**

This cross-sectional, multi-center survey utilized a paper-based questionnaire consisting of 21 questions to capture CM usage and interest as well as CM communication in visceral and thoracic surgical patients being hospitalized at the corresponding departments of surgery at the University Medical Centers in Freiburg, Heidelberg und Ulm, Germany.

**Results:**

Overall, 151 patients consented to the survey. On average, current CM usage was stated by 44% of patients. Most frequently used CM approaches were physical exercise (63%), nutritional supplements (59%) and herbal medicine (56%). Strong interest in CM counselling was stated by 51% of patients. Almost 80% of patients wanted to be treated in a holistic manner and desired for reliable information about CM as well as CM informed physicians. Only 12% of patients communicated CM usage and interest with their attending physician. Review of literature revealed similar results showing an overall CM usage of 43%, preferring nutritional supplements and herbal medicine.

**Conclusion:**

The results of our cross-sectional study indicate a high percentage of CM users and a strong interest in CM among surgical patients. Indeed, the current communication about CM between patients and surgeons is poor. With respect to safety and quality reasons, but also to pay attention to patients’ demands, physicians should be aware of patients’ CM usage in surgery.

**Trial registration:**

German Clinical Trial register (DRKS00015445).

## Introduction

Cross-sectional surveys indicate experience with complementary medicine (CM) of approximately two thirds of the population in Western countries [1–3]. CM consists of different holistic medical approaches such as Traditional Chinese Medicine (TCM), anthroposophic medicine, homeopathy as well as naturopathy. Common therapies of CM are acupuncture, different types of physical exercise, changing of diet and fasting as well as herbal medication. CM is often used independently and without medical consultation [1]. Since the 1970ies frequency of CM usage is increasing, but it depends on patients’ disease as well as on sex and socioeconomic state [4–6]. CM is not only used at home as a previous research work by our group suggested also a high CM usage frequency of almost 50% in hospitalized patients [7]. Interestingly, the results of this study indicated that CM might also play a role in surgery [7]. Studies about CM usage in surgery are widely lacking. Two publications from Canada and Hungary indicate a CM usage frequency of 30% in inpatient general surgical patients [8, 9]. A survey examining German orthopedic and trauma surgical patients showed usage rates of 30%, whereas two thirds of patients stated to be interested in CM [10]. Uncontrolled CM treatment in surgery might be a safety issue due to potential interactions and side effects [8, 11, 12]. Recent research suggests an affection of blood coagulation of some herbal medicine potentially leading to higher risk of bleeding intraoperatively [13, 14]. Communication about CM between patients and treating physicians appears to be poor as only a few patients communicate about CM usage, tightening the safety issue [8–10, 15–18]. Moreover, as patient-physician-contacts in surgery appear to be short and ward rounds just last a few minutes in average [19], it is possible that there is a large gap between patients’ CM needs and communication about it in surgery. Beside risks and safety hazards, CM in surgery might also be a chance to improve for example perioperative management of pain and postoperative paralytic ileus [20–22]. Due to the challenge of a patient-centered therapy respecting the patient’s individual demands, the relevance of CM in surgery has to be addressed by research. To estimate the relevance of CM in surgery, two central hypotheses were posed – (I) a considerable part of hospitalized surgical patients uses CM during their hospital stay and (II) surgical patients are interested in CM, which should be addressed by a subgroup analysis of a multicenter, multidisciplinary cross-sectional study [7].

## Methods

We report about a cross-sectional study. To address the aim of the study, we performed a subgroup analysis of a multicenter, paper-based, multidisciplinary and pseudo-anonymous survey performed at the German University Medical Centers in Freiburg, Heidelberg, Tübingen and Ulm, which was previously reported [7].

### Primary aim of the study

The primary aim of the study was to estimate the relevance of CM in surgery. Two central hypotheses were posed – (I) a considerable part of hospitalized surgical patients uses CM during their hospital stay and (II) surgical patients are interested in CM. Secondary aims of the study were the types of used CM, the communication about CM usage as well as patients’ demands and attitudes towards CM.

### Survey

Only patients being hospitalized at the normal ward at the department of general and visceral surgery (Freiburg und Ulm) and at the department of thoracic surgery (Heidelberg) between April and December 2018 were eligible for participation. In Tübingen, no surgical patients were surveyed. Patients of all ages, regardless of sex, diagnosis and treatment, being able to speak and understand German (at least B2 level according to the European Framework of Reference for Languages [23]) and to complete a questionnaire on their own, were eligible for participation. Recruiting was carried out consecutively. All patients had to give written informed consent before participation. The study was registered at the German Clinical Trial register (DRKS00015445) and approved by the ethical committee of the University Medical Center of Freiburg, Germany (EK FR 25/17) before onset.

### Questionnaire

In absence of a validated questionnaire for surveying CM usage of surgical patients, an existing questionnaire, which previously has been used by the authors, was adjusted [24, 25]. The questionnaire consisted of 21 neutral questions, 15 CM-related and 6 on socio-demographic aspects. Current and previous usage of 21 CM approaches (herbal medicine, balneotherapy, acupuncture/acupressure, mental healing/mindfulness, anthroposophic medicine, homeopathy, physical exercise, aroma therapy, hyperthermia, detoxification, mistletoe, Ayurveda, osteopathy/chiropractic, traditional Chinese medicine (TCM), compresses, colonic cleansing & probiotics, nutritional supplements, diet & nutrition, relaxing/mediation, yoga/qigong, fasting) were evaluated. Furthermore, reasons for and against usage as well as knowledge about CM, interest towards CM and communication about CM usage were inquired. Further information about the questionnaire was previously published [7]. Patients had around 30 min for completing the questionnaires. To avoid manipulation by others, patients had to be able to complete the questionnaire independently and on their own, but they were allowed to ask study staff for help in case of ambiguity. Study staff checked completeness of the questionnaire. In case of incompleteness, the study staff asked the patient to complete the questionnaire independently. The study staff was encouraged to appear friendly but uncommitted.

### Statistics

This was an exploratory subgroup analysis. Based on recent publications, a sample-size of at least 100 patients was calculated to predict reliability of multiple logistic regression including 5 predictive variables (age, male vs. female patients, survey location, nationality and diagnosis of cancer), which are well-known variables affecting CM usage with except of survey location [15, 16]. Beside survey location, all other variables are well-known to affect CM usage [26, 27]. Only patients for whom a complete data set was available were evaluated by multiple logistic regression. The Goodness-of-fit was assessed using the Hosmer-Lemeshow-Test (p < 0.05 was considered significant). Data was entered by three authors in a preformed table and database was closed before onset of analysis (IBM SPSS (Version 27.0)). To analyze distribution and for comparison of categorical variables, chi-squared test was used. P < 0.05 was considered significant. Unless otherwise stated, the results are given as a percentage of patients, who have answered a question, or as absolute values. In case of denial to complete the questionnaire, missing data was not complemented.

## Results

**Fig. 1:**
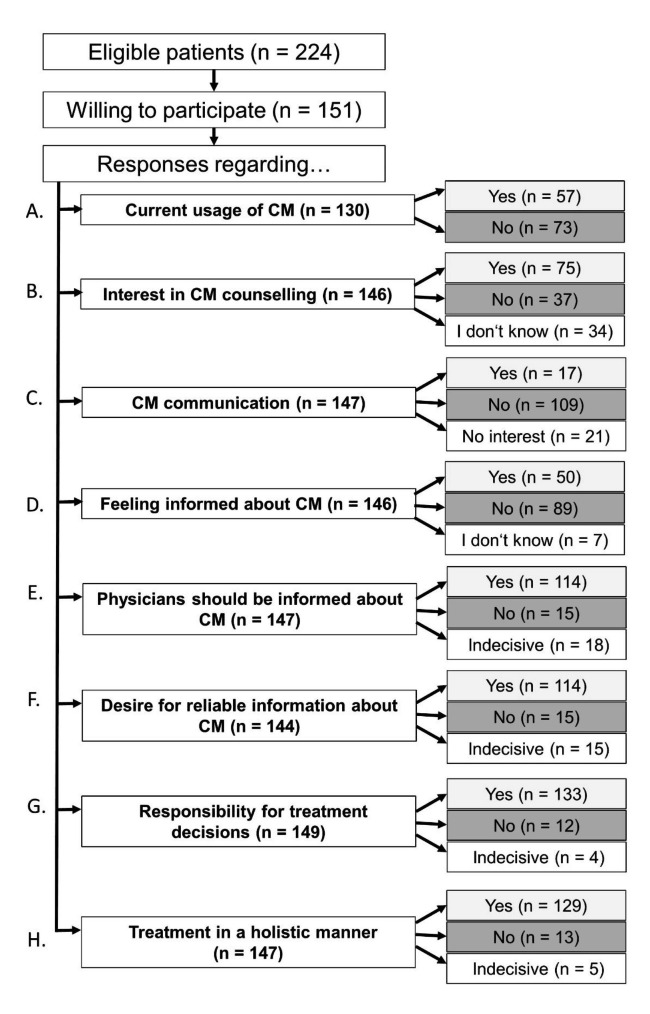
Overview of all included patients, number of responses and results. (CM = Complementary medicine)

Overall, 151 out of 224 eligible patients (67%) were willing to participate. Not all of the participating patients responded to all questions. An overview of all responses including also the number of responding patients is shown in Fig. 1.

Seventy-eight patients (52%) were male and 136 patients (91%) were German citizens. The second most common nationalities were Turkish (2%) and Italian (2%). The average age of participants was 59 ± 15 years (range 18–86 years). One third of the patients (36%) was hospitalized due to cancer.

### CM usage of surgical patients (hypothesis I)

Fifty-seven patients (44%) stated current usage of CM (Fig. 1, A).

**Fig. 2:**
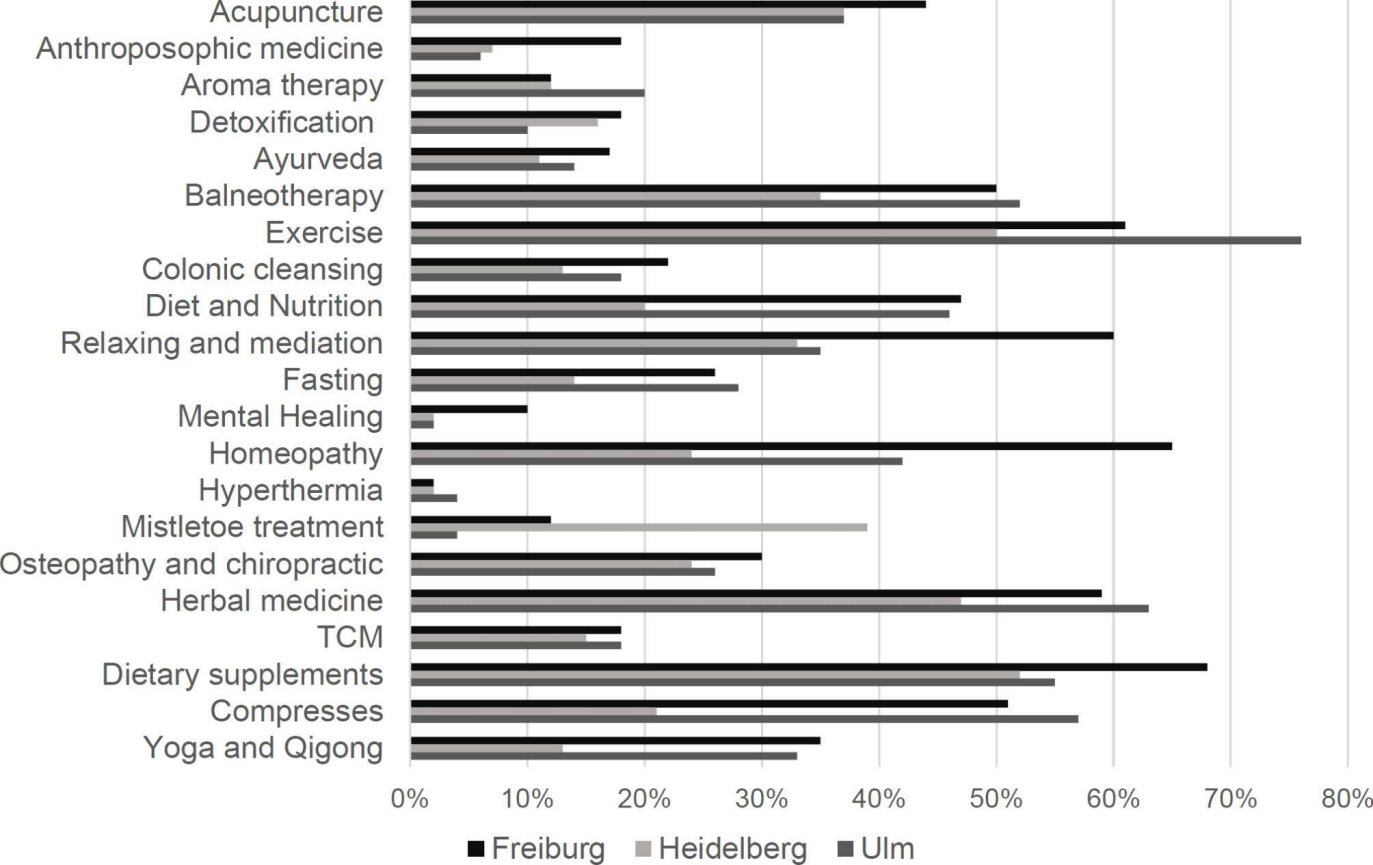
Previously and currently used CM approaches in Freiburg (black bar), Heidelberg (light grey bar) and Ulm (dark grey bar) (Percentage of patients, who replied to the question; TCM = Traditional Chinese Medicine)

Most frequently current or previously used CM approaches were physical exercise (63%), nutritional supplements (59%), herbal medicine (56%), balneotherapy (46%) and homeopathy (45%) (Fig. 2).

### CM interest of surgical patients (hypothesis II)

Strong interest in CM counselling was stated by 75 patients (51%). Further 37 patients (25%) were indecisive (Fig. 1, B).

### Communication about CM in surgical patients

Only 17 patients (12%) stated to communicate CM usage and interest with their attending physicians (Fig. 1, C). Reasons for non-communication were no time (56%), being afraid of physician’s negative attitude towards CM (20%) and missing competence of the physician (16%).

### Further results of survey: demands of patients

Eighty-nine patients (60%) stated to feel little or not at all informed about CM (Fig. 1, D). Reliable information about CM was desired by 114 patients (79%), and 114 patients (78%) stated that physicians should be informed about CM (Fig. 1, E and F). One hundred thirty-three patients (89%) wanted to take over responsibility for treatment decisions, and 129 patients (88%) wanted to be treated in a holistic manner (Fig. 1, G and H).

### Further results of survey: factors affecting CM usage and CM communication

Current usage of CM differed between the locations as patients in Freiburg (71%) had a significantly higher CM usage rate compared to patients in Ulm (22%) and Heidelberg (32%, Table 1). In Freiburg, the most frequently used CM approaches were nutritional supplements (68%), homeopathy (65%) and physical exercise (61%). In Heidelberg, the most frequently used CM approaches were nutritional supplements (52%), physical exercise (50%) and herbal medicine (47%) and in Ulm, physical exercise (76%), herbal medicine (62%) and compresses (57%). Usage frequency of all surveyed CM approaches subdivided by survey location is shown in Fig. 1. The survey location had a significantly influence on CM usage rate (p = 0.001, Table 2) and CM communication (p = 0.048, Table 3).

Significantly more patients in Heidelberg felt little or not at all informed about CM compared to Freiburg und Ulm (80% vs. 60% and 59%, p = 0.020, Table 1). Patients in Heidelberg suffered significantly more frequently from cancer (32% vs. 27% and 20%, p < 0.001), but multiple logistic regression did not reveal an influence of cancer diagnosis on frequency of current CM usage (Table 2).

CM communication was more likely in patients with diagnosis of cancer (p = 0.018, Table 3).

All results of multiple logistic regression considering factors, which might be able to affect CM usage and CM communication are shown in Tables 2 and 3.

**Table 1:** Comparison of results of three University Medical Centers

	Freiburg (n = 52)	Heidelberg (n = 48)	Ulm (n = 51)	p
***Sociodemographic aspects***				
Department of… surgery	General and visceral	Thoracic	General and visceral	**-**
Age (years ± standard deviation)	59.7 ± 14.2	58.0 ± 13.9	59.6 ± 16.3	0.786*
Gender (n male/n female, % male)	28/24, 54%	28/20, 58%	22/29, 43%	0.295
Nationality (n German/n Other, % German)	50/2, 96%	42/6, 88%	44/6, 88%	0.242
Cancer (n yes/ n no/ n not stated, % yes)	14/38, 27%	31/12/5, 38%	10/41, 20%	**< 0.001**
***Usage, interest and communication***				
Current usage of CM (n yes/ n no/n not stated, % yes)	37/15, 71%	9/19/20, 32%	11/39/1, 22%	**< 0.001**
Most frequently used CM approach	Dietary supplements	Dietary supplements	Physical exercise	-
Interested in CM counselling (n yes/ n no/ n I don’t know/ n not stated, % yes)	29/12/9/2, 58%	20/15/11/2, 44%	26/7/17/1, 52%	0.128
Communication about CM (n yes/ n no/n not stated, % yes)	8/44, 15%	3/41/4, 7%	6/44/1, 12%	0.405
***Patients’ requests***				
Treatment in a holistic manner (n yes/ n no/ n I don’t know/ n not stated, % yes)	44/5/2/1, 86%	39/5/1/3, 87%	46/3/2, 90%	0.888
Responsibility for treatment decisions (n yes/ n no/ n I don’t know/ n not stated, % yes)	48/3/1, 92%	40/5/2/1, 85%	45/4/1/1, 90%	0.825
Feel less or even not informed about CM (n yes/ n no/ n I don’t know/ n not stated, % yes)	30/21/1, 60%	35/9/1/3, 80%	24/20/5/2, 59%	**0.020**
Desire for reliable information (n yes/ n no/ n I don’t know/n not stated, % yes)	43/2/3/4, 90%	31/7/8/2, 67%	40/6/4/1, 80%	0.106
Physician should be informed about CM (n yes/ n no/ n I don’t know/ n not stated, % yes)	38/4/8/2, 76%	34/8/5/1, 72%	42/3/5/1, 84%	0.334

(Percentage of all responding patients; p-values calculated by chi-squared-test, *p-value calculated by Kruskal-Wallis-test)

**Table 2:** Factors affecting usage of complementary medicine

Parameter	Regression coefficient	Standard error	p*	Odds ratio	95%-Confidence Interval	
					**Lower**	**Upper**
Age	0.002	0.016	0.890	1.002	0.972	1.033
*Sex* Male Female	Reference -0.297	Reference 0.436	Reference 0.496	Reference 0.743	Reference 0.316	Reference 1.747
*Location* Freiburg Ulm Heidelberg	Reference 2.151 2.169	Reference 0.470 0.651	Reference **< 0.001** **0.001**	Reference 8.590 8.754	Reference 3.417 2.443	Reference 21.596 31.361
*Cancer* Yes No	-0.505 Reference	0.519 Reference	0.330 Reference	0.603 Reference	0.218 Reference	1.669 Reference
*Nationality* German Other	Reference 0.422	Reference 0.772	Reference 0.585	Reference 1.524	Reference 0.335	Reference 6.927

*Multiple logistic regression, highest sample-size group was chosen as reference.

Goodness-of-fit was assessed using the Hosmer-Lemeshow-Test, indicating a good model fit, χ²(8) = 4.670, p = 0.792.

Only patients for whom a complete data set was available were evaluated (n = 125).

**Table 3:** Factors affecting communication about complementary medicine

Parameter	Regression coefficient	Standard error	p*	Odds ratio	95%-Confidence Interval	
					**Lower**	**Upper**
Age	0.11	0.020	0.591	1.011	0.973	1.050
*Sex* Male Female	Reference -0.803	Reference 0.599	Reference 0.180	Reference 0.448	Reference 0.138	Reference 1.449
*Location* Freiburg Ulm Heidelberg	Reference 0.170 1.750	Reference 0.627 0.886	Reference 0.787 **0.048**	Reference 1.185 5.757	Reference 0.347 1.014	Reference 4.051 32.677
*Cancer* Yes No	Reference -1.467	Reference 0.621	Reference **0.018**	Reference 0.231	Reference 0.068	Reference 0.779
*Nationality* German Other	Reference 0.312	Reference 1.141	Reference 0.784	Reference 1.367	Reference 0.146	Reference 12.778

*Multiple logistic regression, highest sample-size group was chosen as reference.

Goodness-of-fit was assessed using the Hosmer-Lemeshow-Test, indicating a good model fit, χ²(8) = 5.427, p = 0.711.

Only patients for whom a complete data set was available were evaluated (n = 141).

## Discussion

This is the first survey assessing CM usage and interest amongst patients hospitalized at surgical departments of University Medical Centers in Germany. We found almost half of the patients stating a current CM usage during their hospital stay; physical exercise, herbal medicine and dietary supplements were most frequently used.

Similar to the results of our survey, literature review revealed an average CM usage frequency of approximately 43% in surgical patients of all disciplines (Table 4). CM usage of surgical patients varies internationally between 16 and 75%, depending among others on country and definition of CM. A variety of surveys indicate a higher CM affinity of women [8, 10, 28–30], but we found no influence of sex on CM usage frequency. As shown in Table 4, recent research suggests that the focus of patient-selected CM is on herbal medicine and dietary supplements, which is also confirmed by the results of our survey. Patients often consider CM, especially herbal medicine and dietary supplements, as safe and harmless [10, 31, 32]. The risk of CM interventions, especially of herbal medicine and dietary supplements, is still a matter of scientific discussion. Herbal medicine and dietary supplements may cause side-effects or interactions, but evidence is mostly lacking and serious CM-related risks appear to be at least unlikely [33–36]. Potential interactions being relevant in surgery are effects on blood coagulation as wells as anesthesia-related risks such as arrhythmogenic potential and interactions with membrane receptors. Recent literature recommends to stop intake of herbal medication and dietary supplements with a related risk profile before surgery [11, 21, 37]. On the other hand, CM might improve self-management and patient-centered care and offers promising approaches for the treatment of typical postoperative complaints such as nausea and vomiting, pain and sleep disturbances which might be integrated into daily surgical practice [21]. Recent surveys indicate that approximately two thirds of surgical patients in Western countries are interested in CM (Table 4). Almost 90% of patients in our study and also almost 90% of patients in a prior cross-sectional study evaluating orthopedic patients in Germany showed that patients attached importance to make their own treatment decisions [10]. The integration of a patient’s view for decision making and the usage of shared decision making model is known to improve treatment adherence, knowledge of patients and patients satisfaction [38–40]. The high rate of patients desiring to take over responsibility for treatment decisions also supports the important role of self-management. Self-management is a central element of CM and an effective approach to improve treatment of chronic diseases [41, 42]. Unfortunately, the communication about CM between patients and attending surgeons is poor (Table 4), and similarly low communication rates are reported for other physicians such as oncologists and radiologists [18, 29, 43]. In our study, the most stated reason for non-communication of CM usage or interest was “no time”. In general, this is not surprising as research about communication in surgery indicates that patients report the feeling of lack of information and too less time for deeper communication with their attending surgeons [44, 45]. Nevertheless, the statement of “no time for communication” emphasizes that CM was not considered as relevant enough for communication. Similarly, parents of children suffering from cancer responded that the reason for non-communication was that no one raised the question about CM [46]. As mentioned above, patients consider CM as harmless, which is why, patients might be not able to see the clinical relevance of CM usage in surgery. It is, therefore, a physician’s task to ask for CM. Furthermore, a fifth of patients in our study stated that they were afraid of the physician’s attitude towards CM. In a cross-sectional study by Stub et al., who evaluated the attitudes and knowledge of health care providers regarding CM in cancer care, it was shown that most.

**Table 4:** Review of recent literature regarding in-patient surgical patients and frequency of current complementary medicine usage, interest and most frequently used complementary medicine approach as well as communication with the attending surgeons

Publication	Year	Origin	Patients	Type of surgery	Frequency of CM…			Most frequently used CM
					**Usage**	**Interest**	**Communication**	
This study	2021	Germany	Mixed adults	General, thoracic & visceral	44%	51%	12%	Physical exercise
Adusumilli et al. [49]	2004	USA	Mixed adults	Mixed	16%^*^	-	7%^*^	Herbal medicine^*^
Andersen et al. [50]	2015	USA	Women with cancer	Breast cancer	29%	-	83%^+^	Dietary supplements
Braun et al. [51]	2011	Australia	Mixed adults	Cardiac	42%	85%	44%	Dietary supplements
Dalmayrac at al. [52]	2016	France	Mixed adults	Cardiac	39%^°^	-	29%	Physical therapies
Dhanoa et al. [53]	2014	Malaysia	Mixed with cancer	Orthopedic	61%^°^	-	31%	Herbal medicine
Guilmetdinov et al.[54]	2019	Australia	Mixed adults	Mixed	44%	-	86%^+^	Dietary supplements
Kilper et al. [10]	2020	Germany	Mixed adults	Orthopedic & trauma	30%	65%	15%	Physical exercise
Lim et al. [55]	2010	Singapore	Adults with cancer	Otolaryngology-head & neck	68%	-	-	Herbal medicine
Lin et al. [56]	2004	USA	Children	Pediatric	30%^°^	-	-	Herbal medicine
Liu et al.	2000	USA	USA	Cardiac	75%	-	17%	Dietary supplements
Norred [57]	2002	USA	Mixed adults	Mixed	67%	-	-	Dietary supplements
Schiemann et al. [9]	2009	Canada	Mixed adults	Visceral	27%	-	-	Herbal medicine
Shakeel et al. [33]	2008	Scotland	Mixed adults	General surgery	46%^°^	-	40%	Herbal medicine
Shakeel et al. [58]	2009	Scotland	Mixed adults	Otolaryngology-head & neck	36%	-	43%	Herbal medicine
Soos et al. [8]	2016	Hungary	Mixed adults	General & visceral	27%	64%	13%	Acupuncture
Wang et al. [59]	2003	USA	Mixed adults	Mixed	25%	-	45%	Self-prayer
Yazici et al. [60]	2018	Turkey	Mixed adults	Mixed	66%	-	-	Herbal medicine
Yoshimura et al. [61]	2003	Japan	Men with cancer	Prostatectomy/radiation	20%	-	-	Herbal medicine
**Overall**					**43%** (without */°)	**66%**	**29%** (without */^+^)	**Herbal medicine**

*Survey focused on herbal medicine, results of other complementary medicine approaches were not reported in detail; °Questionnaire captured usage in the preceding year before surgery; ^+^Patients stated communication with their family doctor. Communication between attending surgeon and patient was not measured.

of the non-CM-skilled responders stated that CM can cause adverse events in cancer treatment, and that they would neither encourage nor discourage the usage of CM in cancer patients [47]. Indeed, this is interesting as CM is rated as risky by the physicians in the study, but no recommendation regarding CM usage is given for the patient. Keeping this in mind, it is also assumable that patients did not communicate their CM usage or interest to their attending physicians as they are not expecting constructive feedback. Recent research indicates an increase of internet searching for health specific topics, although the quality of information is often low [48]. On this way, the low communication rate between physicians and patients might be able to promote uncontrolled CM usage as patients are misled by online-communicated misinformation about CM. Once more, the consequence is an increased risk of side effects and interactions by CM. The non-communication about CM is, therefore, a vicious circle. Attending physicians should be aware of patients’ non-communication, focusing on consider CM usage during anamnesis and treatment. The results of our study emphasize the role of CM in surgery indicating that surgeons should be also informed about CM. Almost 80% of patients in our study stated that attending physicians should have knowledge about CM, which is also confirmed by other studies showing similar results [10]. The demand of patients for reliable information about CM is also a call for science as evidence-based CM is still needed.

### Limitations and strengths

Results of surveys are always limited due to the possibility of selection bias. It is assumable that patients, who are interested in CM, are more interested in participating in a CM-related survey. As previously mentioned, the results of this manuscript are a subgroup analysis of an interdisciplinary survey evaluating CM usage of hospitalized patients in University Medical Centers in Southern Germany, which showed a response rate of 67% [7]. A response rate of approximately 60% is recommended to avoid bias [62]. Furthermore, CM usage was more likely in Freiburg indicating the previously reported limited transferability of surveys due to regional factors such as local culture and institutions [2]. In 2020, Li et al. stated that nature connectedness is a factor for favoring herbal medicine [63]. Freiburg can be characterized as “green city” emphasizing a close affinity to nature and high relevance of nature medicine of the population. The center for complementary medicine in Freiburg (UZN) is one of the first (founded 1998) and largest (> 4000 out-patient and in-patient consultations per year) of its kind in Germany. Even though patients participating in the survey have not been counselled by UZN-physicians during their hospital stay, CM might have a good reputation in Freiburg due to the existence of this center. The size of the three cities (Freiburg, Ulm and Heidelberg) and their socio-economic situation is similar and probably not a relevant factor.

## Conclusion

Our study shows a high percentage of CM users and a strong interest in CM among surgical patients admitted to surgical wards of university hospitals in the South-West of Germany. The complementing review of literatures supports a worldwide CM usage of surgical patients. Current communication about CM between patients and surgeons is poor. The results emphasize the necessity for physicians to be aware of complementary medicine usage, also in surgery. With respect to a patient-centered treatment, but also for safety and quality reasons, the topic is of relevance for all physicians as well as for researchers to promote an evidence-based complementary medicine, which is demanded by patients.

## Data Availability

The datasets used and analysed during the current study are available from the corresponding author on reasonable request.

## Declarations

All methods were performed in accordance with the Declaration of Helsinki.

## Abbreviations

AZKIM: Akademisches Zentrum für Komplementäre und Integrative Medizin.
CM: Complementary medicine.
TCM: Traditional Chinese Medicine.
UZN: Center for complementary medicine Freiburg.
