# Correction: Complementary medicine usage in surgery: a cross-sectional survey in Germany

**DOI:** 10.1186/s12906-022-03772-1

**Published:** 2022-11-19

**Authors:** Ann-Kathrin Lederer, Yvonne Samstag, Thomas Simmet, Tatiana Syrovets, Roman Huber

**Affiliations:** 1 Center for Complementary Medicine, Department of Medicine II, Faculty of Medicine, Medical Center-University of Freiburg, University of Freiburg, Hugstetter Straße 55 – Haus Frerichs, 79106 Freiburg, Germany; 2 Department of General, Visceral and Transplantation Surgery, University Medical Center, Mainz, Germany; 3 Institute of Immunology, Section Molecular Immunology, University Hospital of Heidelberg, Heidelberg, Germany; 4 Institute of Pharmacology of Natural Products & Clinical Pharmacology, Ulm University, Ulm, Germany

**Correction: BMC Complement Med Ther 22, 263 (2022)**

**https://doi.org/10.1186/s12906-022-03746-3**

Following publication of the original article [1], the authors reported an error in Tables 2 and 3. The correct presentation of these tables are given below.

**Table 2:** Factors affecting usage of complementary medicine

Parameter	Regression coefficient	Standard error	p^a^	Odds ratio	95%-Confidence Interval	
					Lower	Upper
Age	0.002	0.016	0.890	1.002	0.972	1.033
*Sex*						
Male	Reference	Reference	Reference	Reference	Reference	Reference
Female	-0.297	0.436	0.496	0.743	0.316	1.747
*Location*						
Freiburg	Reference	Reference	Reference	Reference	Reference	Reference
Ulm	2.151	0.470	**<0.001**	8.590	3.417	21.596
Heidelberg	2.169	0.651	**0.001**	8.754	2.443	31.361
*Cancer*						
Yes	-0.505	0.519	0.330	0.603	0.218	1.669
No	Reference	Reference	Reference	Reference	Reference	Reference
*Nationality*						
German	Reference	Reference	Reference	Reference	Reference	Reference
Other	0.422	0.772	0.585	1.524	0.335	6.927

Goodness-of-fit was assessed using the Hosmer-Lemeshow-Test, indicating a good model fit, χ^2^(8) = 4.670, *p* = 0.792

Only patients for whom a complete data set was available were evaluated (*n* = 125)

^a^Multiple logistic regression, highest sample-size group was chosen as reference

**Table 3:** Factors affecting communication about complementary medicine

Parameter	Regression coefficient	Standard error	p^a^	Odds ratio	95%-Confidence Interval	
					Lower	Upper
Age	0.11	0.020	0.591	1.011	0.973	1.050
*Sex*						
Male	Reference	Reference	Reference	Reference	Reference	Reference
Female	-0.803	0.599	0.180	0.448	0.138	1.449
*Location*						
Freiburg	Reference	Reference	Reference	Reference	Reference	Reference
Ulm	0.170	0.627	0.787	1.185	0.347	4.051
Heidelberg	1.750	0.886	**0.048**	5.757	1.014	32.677
*Cancer*						
Yes	Reference	Reference	Reference	Reference	Reference	Reference
No	-1.467	0.621	**0.018**	0.231	0.068	0.779
*Nationality*						
German	Reference	Reference	Reference	Reference	Reference	Reference
Other	0.312	1.141	0.784	1.367	0.146	12.778

Goodness-of-fit was assessed using the Hosmer-Lemeshow-Test, indicating a good model fit, χ^2^(8) = 5.427, *p* = 0.711

Only patients for whom a complete data set was available were evaluated (*n* = 141)

^a^Multiple logistic regression, highest sample-size group was chosen as reference

In the reference list, reference information below that was indicated in Table 4 has been added.

Liu EH, Turner LM, Lin SX, Klaus L, Choi LY, Whitworth J, et al. Use of alternative medicine by patients undergoing cardiac surgery. J Thorac Cardiovasc Surg. 2000;120:335–41.

The original article [1] has been updated.
